# Noise-robust temporal–spectral fusion transformers for EEG-based cognitive state classification in aviation environments

**DOI:** 10.3389/fdata.2026.1837706

**Published:** 2026-07-09

**Authors:** Quynh Anh Nguyen, Nam Anh Dao, Long Nguyen

**Affiliations:** 1 Faculty of Information Technology, Electric Power University, Ha Noi, Vietnam; 2 Computer Science and Engineering, Speed School of Engineering, University of Louisville, Louisville, KY, United States

**Keywords:** Attention-related Pilot Performance Decrements (APPD), aviation safety analytics, cognitive state classification, electroencephalography (EEG), multimodal feature learning, noise-robust machine learning, temporal–spectral fusion, transformer networks

## Abstract

Attention-related Pilot Performance Decrements (APPD) contribute substantially to aviation incidents, yet existing electroencephalography (EEG)-based monitoring methods often lack generalization, robustness to noise, and effective temporal–spectral integration. We propose a temporal–spectral fusion transformer (TF-T) combining multi-scale preprocessing, dual-stream temporal and spectral feature extraction, and transformer-based fusion with enhanced temporal–spectral integration and multi-resolution feature processing for multiclass cognitive state recognition. Three variants (TF-T1–TF-T3) are evaluated on controlled and ecologically realistic EEG datasets under clean and noise-augmented (Gaussian, Uniform, COMBO) conditions, using chronological partitioning to avoid temporal leakage. TF-T2 achieves the highest clean-data accuracy (99.2%), while TF-T3 offers superior robustness, improving Macro-F1 by ~4.5–4.7 points across all noise types and outperforming state-of-the-art baselines by up to +8 Macro-F1 under COMBO noise, supporting its deployment in perturbation-prone aviation environments.

## 1 Introduction

Despite substantial advances in aviation technology and automation, human performance remains a critical determinant of flight safety. Statistics indicate that more than 75% of pilot errors stem from perceptual failures (Jones and Endsley, 1996), highlighting that even highly trained professionals are susceptible to dangerous lapses in attention. These lapses are not random; rather, they follow distinct and recurrent patterns identified in accident investigations. According to the International Air Transport Association (IATA), between 2012 and 2021, there were 45 accidents caused by pilots losing control of the aircraft, resulting in 1,645 fatalities (International Air Transport Association, 2022, 2019). Furthermore, the Commercial Aviation Safety Team (CAST) found that in 16 out of 18 major accidents, attention deficiencies played a role (Commercial Aviation Safety Team, n.d.). Three particularly hazardous Attention-related Pilot Performance Decrements (APPD) have been identified: *Channelized Attention* (CA), where fixation on a single task causes neglect of other critical information; *Diverted Attention* (DA), in which competing tasks fragment situational awareness; and *Startle/Surprise* (SS), where unexpected events induce cognitive paralysis (Yen et al., 2009; Hankins and Wilson, 1998; Boksem and Tops, 2008).

Electroencephalography (EEG) has emerged as a promising modality for detecting such cognitive states before they manifest in overt behavior. Its millisecond temporal resolution allows monitoring of transient neural activity changes indicative of attention deficits (Bigdely-Shamlo et al., 2015; Fló et al., 2022). However, EEG signals are inherently noisy, prone to contamination from environmental and physiological artifacts, and exhibit high inter-subject variability–challenges compounded in real-world aviation environments.

### 1.1 Prior work in EEG-based pilot state monitoring

Early EEG-based studies relied on manual preprocessing techniques, including band-pass filtering to isolate frequency bands such as beta rhythms (Roza and Postolache, 2019) or broader ranges (0.1—50 Hz) before applying Independent Component Analysis (ICA) (Han et al., 2020). Yet, comparative studies have found that ICA does not always yield significant gains over filtering (Alreshidi et al., 2022). This motivated the development of automated artifact rejection, with *Autoreject* (Jas et al., 2017)–successfully applied in Bonassi et al. (2021); Pousson et al. (2021)–offering adaptive, channel-specific thresholding and repair.

Feature extraction methods have evolved from basic statistical measures (Harrivel et al., 2017; Hasan et al., 2020) to more complex approaches such as wavelet packet transforms for brain-wave decomposition (Wu et al., 2019) and common spatial pattern filtering (Binias et al., 2018). Beta-wave metrics have proven particularly effective: Ji et al. (2024) analyzed beta energy ratios and Shannon entropy during turning phases, achieving up to 93.67% test accuracy, while Feng et al. (2024) found beta sub-band power (16—30 Hz) to be a robust indicator of situation awareness, reaching 92% classification accuracy.

Riemannian geometry-based methods (Barachant et al., 2012; Majidov and Whangbo, 2019) have further enhanced EEG representation by mapping covariance matrices to the tangent space of a Riemannian manifold, improving generalization across subjects. However, such methods require computationally expensive operations (e.g., matrix logarithms), limiting real-time applicability.

Deep learning approaches have expanded capability by learning joint temporal–spatial patterns. Johnson et al. (2015) benchmarked traditional classifiers, while Han et al. (2020) combined CNN and LSTM modules to detect distraction, workload, fatigue, and normal states. Wu et al. (2019) employed deep contractive autoencoders for fatigue detection, and Harrivel et al. (2016) explored ensembles for predicting APPD states. Nonetheless, many of these methods are constrained to binary or limited multiclass settings, lack integrated temporal–spectral modeling, or underperform under noise.

### 1.2 Alternative sensing modalities

Eye-tracking has been used to quantify pilot workload, with studies such as Cheng et al. (2024) showing changes in fixation patterns and pupil diameter during emergency procedures. Facial recognition systems (Samy et al., 2025) detect fatigue via facial landmarks and expression analysis, achieving up to 96.8% accuracy under ideal conditions. While these modalities are non-invasive, they cannot directly capture latent neural states and are sensitive to environmental factors.

### 1.3 Dataset foundations

This study leverages two complementary datasets that jointly enable evaluation under high-density controlled conditions and ecologically realistic scenarios.

**Dataset A: Reducing Commercial Aviation Fatalities** (Kaggle, n.d.) contains multimodal physiological recordings from 18 professional pilots (nine two-person crews) performing tasks designed to elicit CA, DA, and SS states. Signals include 22-channel EEG (256 Hz), ECG, respiration, and galvanic skin response (GSR). Data are chronologically partitioned into training (56%), validation (8.4%), and test (35.6%) sets to prevent temporal leakage. Four variants—*BASE, GAUSS, RAND*, and *COMBO*—simulate real-world noise.

**Dataset B: Flight-Deck Mental Workload (FDMW)** (Hernández-Sabaté et al., 2024) was recorded with a portable 14-channel EEG system (128 Hz) across three scenarios: *N-back* (BL, LW, MW, HW), *Heat-the-Chair* (BL, LW, HW), and *Flight Simulator* (BL, E, M, MH, H). Scenario-specific splits avoid temporal leakage, and the same four noise variants are applied while preserving class balance.

### 1.4 Research gaps

Despite progress, current EEG-based aviation monitoring systems face persistent issues: (i) limited ability to generalize across pilots and environments; (ii) inadequate integration of temporal evolution and spectral structure; (iii) narrow state coverage, often excluding the full APPD spectrum; (iv) sensitivity to noise; and (v) computational demands that hinder real-time deployment. Existing methods frequently require extensive calibration and often lack interpretability–an essential requirement for safety-critical adoption.

### 1.5 Contributions

The primary contributions of this work are as follows:

- We introduce a *temporal—spectral fusion transformer* (TF-T) architecture tailored for EEG-based multiclass cognitive state recognition in aviation contexts. The framework integrates multi-scale preprocessing, dual-stream temporal and spectral feature extraction, and enhanced temporal–spectral fusion framework to jointly capture cross-frequency relationships and inter-channel dependencies.
- We design a progressive architecture evolution across three configurations (TF-T1, TF-T2, TF-T3), systematically incorporating enhancements such as progressive architectural refinements including multi-resolution temporal processing and enhanced temporal–spectral feature integration to address robustness under real-world noise conditions.
- We establish a rigorous, ecologically valid evaluation protocol using two complementary datasets—Dataset A (high-density, controlled elicitation of CA, DA, SS) and Dataset B (portable EEG, realistic workload scenarios across N-back, HtC, and Flight Simulator tasks)—with multiple noise-augmented variants to simulate operational perturbations.
- We provide a comprehensive analysis of classification behavior, including per-class sensitivity, robustness curves under graded noise levels, and an error taxonomy that distinguishes benign from harmful misclassifications, offering interpretable insights for operational deployment.

### 1.6 Performance highlights

In extensive experiments, TF-T2 achieves the highest clean-data accuracy (99.2% on Dataset A), while TF-T3 delivers consistent robustness advantages under all noise types, improving Macro-F1 by approximately 4.5—4.7 points compared to TF-T2. Against state-of-the-art baselines, TF-T3 improves Macro-F1 by up to +8 points under COMBO noise and reduces temporal instability by ≈35%.

### 1.7 Paper structure

The remainder of this paper is organized as follows: Section 2 details the proposed method; Section 3 describes the datasets; Section 4 presents experimental design choices; Section 5 discusses findings, limitations, and implications; and Section 6 concludes the work.

## 2 Proposed method

**Figure 1:**
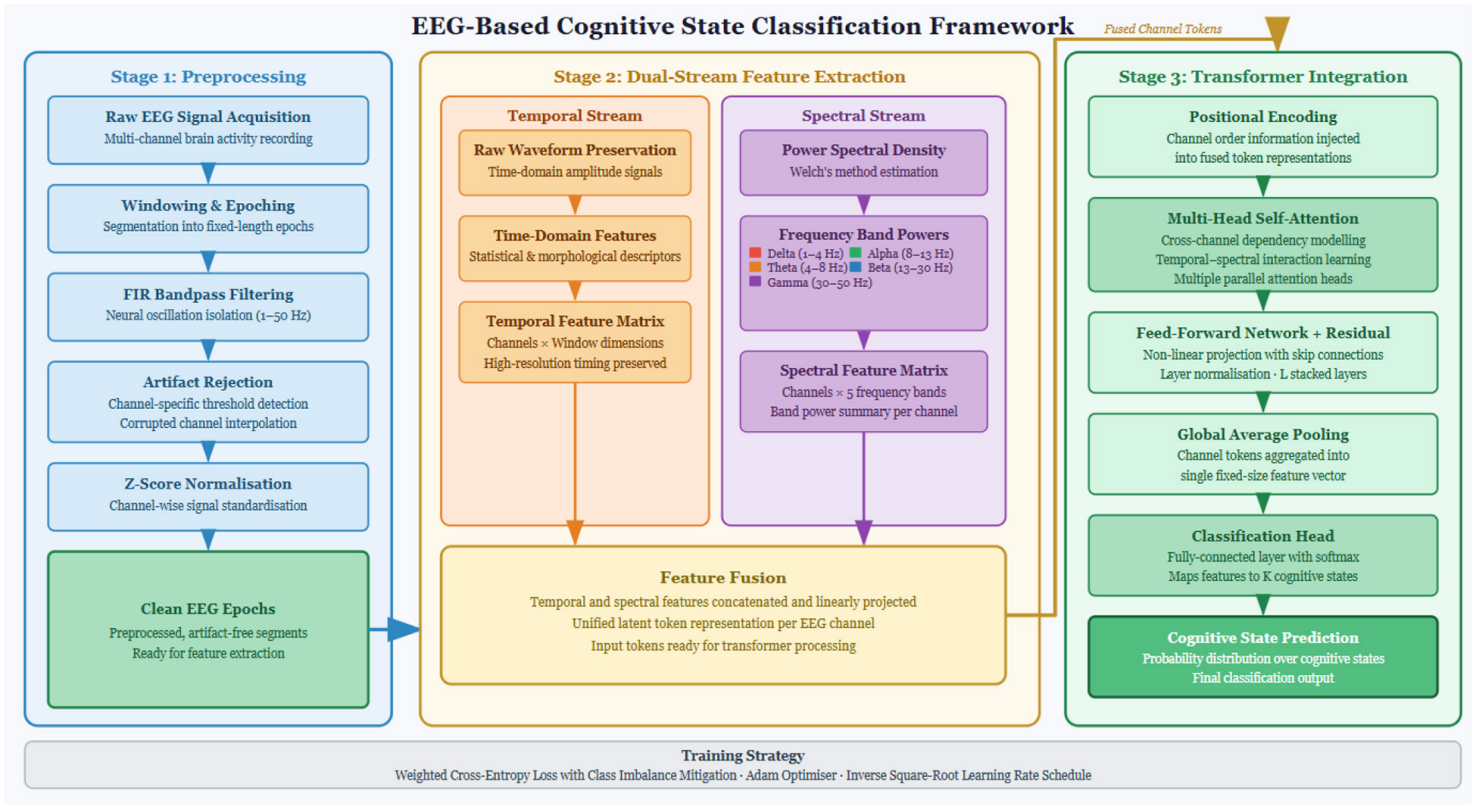
Overview of the proposed temporal–spectral fusion pipeline for EEG-based cognitive state classification.

We propose a three-stage framework for EEG-based cognitive state classification that jointly models temporal and spectral neural dynamics via a transformer encoder. The architecture is specifically designed to address two major challenges in this domain: (*i*) the high susceptibility of EEG to non-neural artifacts, and (*ii*) class imbalance arising from the unequal prevalence of cognitive states. The framework operates as follows: *Stage 1* applies multi-scale preprocessing to suppress artifacts while preserving physiological rhythms; *Stage 2* extracts complementary time- and frequency-domain descriptors and projects them into a unified latent representation; and *Stage 3* integrates these features using multi-head self-attention to capture cross-channel dependencies and modality interactions. The complete workflow is illustrated in Figure 1. Equations 1–44 will describe the proposed models mathematics.

### 2.1 Notation and conventions

All variables and symbols used in the following sections are listed in Table 1. Where a symbol is reused in different contexts, its meaning is explicitly specified to avoid ambiguity.

**Table 1:** Notation and conventions used in the proposed method.

Symbol	Definition
*C*	Number of EEG channels
*T*	Number of time samples in the continuous recording
**X**∈ℝ^*C*×*T*^	Raw multichannel EEG (channels × time)
*w*	Epoch length (samples)
${\text{E}}_{i}\in {\mathbb{R}}^{C\times w}$	*i*-th EEG epoch
*h*[*n*], *L*	FIR filter impulse response and its length
θ_*c*_	Learned artifact-rejection threshold for channel *c*
D(·,·)	Artifact indicator function, returning 0 or 1
$N_{c}$	Set of spatial neighbors of channel *c*
*w*_*j*_ (spatial)	Spatial interpolation weight for neighbor *j*; sums to 1; *not* related to class weighting
μ_*c*_, σ_*c*_	Mean and standard deviation of channel *c* (per epoch unless stated)
*M*, *R*, *K*	Welch PSD parameters: segment length, hop size, and number of segments
*v*[*n*]	Windowing function (e.g., Hamming)
*f*, Δ*f*	Discrete frequency bin and resolution
*b*	Frequency band index in {δ, θ, α, β, γ}
$\left[f_{\min}^{b},f_{\max}^{b}\right]$	Frequency range of band *b*
${\text{B}\text{P}}_{i}^{c,b}$	Band power of channel *c* in band *b*
${\text{F}}_{i}^{temp}\in {\mathbb{R}}^{C\times w}$	Temporal-domain features
${\text{F}}_{i}^{spec}\in {\mathbb{R}}^{C\times 5}$	Spectral-domain features
${\text{F}}_{i}^{fused}\in {\mathbb{R}}^{C\times d_{model}}$	Fused temporal–spectral features
**W**_*fuse*_, **b**_*fuse*_	Linear fusion weights and bias
*S*	Transformer sequence length (*S* = *C*; tokens are channels)
*d*_*model*_	Transformer model dimension
*H*, *d*_*k*_, *d*_*v*_	Number of attention heads, key dimension, and value dimension per head
$\text{P}\text{E}\in {\mathbb{R}}^{C\times d_{model}}$	Positional encoding matrix
*l* = 1, …, *L*	Transformer layer index
${\text{W}}_{h}^{Q},{\text{W}}_{h}^{K},{\text{W}}_{h}^{V}$	Query, key, and value projections for head *h*
**W** ^*O*^	Multi-head output projection
**W**_*cls*_, **b**_*cls*_	Classification head weights and bias
*K*	Number of output classes
*N*	Number of training samples
*y*_*i, j*_	One-hot label for sample *i*, class *j*
*p*_*i, j*_	Predicted probability for sample *i*, class *j*
*N*_*j*_	Number of samples in class *j*
*w*_*j*_ (class)	Class weight for loss balancing; computed from inverse class frequency; *not* related to spatial interpolation
*step*, *warmup*_*steps*	Training step and warmup length in the learning rate schedule

Symbols with multiple contextual meanings are explicitly described.

### 2.2 Stage 1: multi-scale pre-processing

The first stage ensures that the raw EEG is free from major artifacts and standardized across channels, forming a stable basis for feature extraction. The continuous EEG recording **X** is segmented into non-overlapping one-second epochs. Let *f*_*s*_ denote the sampling frequency and *w* = *f*_*s*_ the epoch length in samples. The *i*-th epoch is defined as


$$\begin{array}{l} {\text{E}}_{i}=\text{X}\left[:,,i\cdot w:\left(i+1\right)\cdot w\right],\;i=0,1,\ldots ,\left\lfloor \frac{T}{w}\right\rfloor -1, \end{array}$$ (1)

yielding temporally localized EEG segments that preserve the original temporal structure while avoiding redundancy between adjacent epochs.

Each epoch undergoes FIR bandpass filtering to retain neural oscillations between 1–50 Hz:


$$\begin{array}{l} {\text{E}}_{i}^{f}\left[c,n\right]=\overset{L-1}{\underset{k=0}{\sum }}h\left[k\right]\cdot {\text{E}}_{i}\left[c,n-k\right], \end{array}$$ (2)

thus removing slow drifts and high-frequency noise without distorting relevant rhythms.

Artifact rejection is then applied using *Autoreject*, which learns channel-specific thresholds θ_*c*_ and flags corrupted segments:


$$\begin{array}{l} D\left({\text{E}}_{i}^{f}\left[c,:\right],\theta_{c}\right)=\{\begin{array}{ll} 1, & \max\left(\left|{\text{E}}_{i}^{f}\left[c,:\right]\right|\right)>\theta_{c},\\ 0, & \text{otherwise}. \end{array} \end{array}$$ (3)

Corrupted channels are reconstructed by spherical spline interpolation from spatial neighbors:


$$\begin{array}{l} {\text{E}}_{i}^{r}\left[c,n\right]=\{\begin{array}{ll} \sum_{j\in N_{c}}w_{j}\cdot {\text{E}}_{i}^{f}\left[j,n\right], & D=1,\\ {\text{E}}_{i}^{f}\left[c,n\right], & \text{otherwise}, \end{array} \end{array}$$ (4)

with $\underset{j}{\sum }w_{j}=1$.

Finally, z-score normalization is performed channel-wise:


$$\begin{array}{l} {\text{E}}_{i}^{norm}\left[c,:\right]=\frac{{\text{E}}_{i}^{r}\left[c,:\right]-\mu_{c}}{\sigma_{c}}, \end{array}$$ (5)

ensuring amplitude comparability across channels and epochs.

The FIR filter was designed using a linear-phase windowed-sinc formulation with passband limits of 1–50 Hz. Artifact rejection was performed using the Autoreject framework with channel-specific threshold estimation. Channel normalization was applied independently for each channel using z-score standardization. To prevent information leakage, preprocessing parameters were estimated using training data and subsequently applied to validation and test partitions.

### 2.3 Stage 2: dual-stream feature extraction and fusion

The second stage derives two complementary feature sets. The *temporal stream* directly preserves normalized waveforms:


$$\begin{array}{l} {\text{F}}_{i}^{temp}={\text{E}}_{i}^{norm}. \end{array}$$ (6)

The *spectral stream* captures frequency-specific power distributions via Welch's method:


$$\begin{array}{l} {\text{P}}_{i}^{c}\left(f\right)=\frac{1}{K}\overset{K-1}{\underset{j=0}{\sum }}|\overset{M-1}{\underset{n=0}{\sum }}{\text{E}}_{i}^{norm}\left[c,jR+n\right]v\left[n\right]e^{-i2\pi fn/M}{|}^{2}. \end{array}$$ (7)

Band powers are obtained by summing over canonical frequency ranges:


$$\begin{array}{l} {\text{B}\text{P}}_{i}^{c,b}=\underset{f\in \left[f_{\min}^{b},f_{\max}^{b}\right]}{\sum }{\text{P}}_{i}^{c}\left(f\right)\cdot \Delta f, \end{array}$$ (8)

forming the spectral feature matrix ${\text{F}}_{i}^{spec}$.

Temporal and spectral features are concatenated channel-wise and projected into a shared latent space:


$$\begin{array}{l} {\text{F}}_{i}^{fused}={\text{W}}_{fuse}\cdot \left[{\text{F}}_{i}^{temp};{\text{F}}_{i}^{spec}\right]+{\text{b}}_{fuse}. \end{array}$$ (9)

This produces *C* channel tokens, each of dimension *d*_*model*_, aligned for joint modeling.

Although Dataset A and Dataset B differ in channel count and sampling frequency, the preprocessing and feature extraction stages generate fixed-dimensional embeddings before transformer processing. Consequently, the transformer backbone remains unchanged across datasets, while only the input representation dimensions are adapted during feature encoding.

### 2.4 Stage 3: transformer-based temporal–spectral integration

The third stage integrates the fused channel tokens using a transformer encoder. To encode channel ordering, sinusoidal positional embeddings are added:


$$\begin{array}{ll} \text{P}\text{E}\left(pos,2i\right) & =\sin\left(\frac{pos}{10000^{2i/d_{model}}}\right), \end{array}$$ (10)

$$\begin{array}{ll} \text{P}\text{E}\left(pos,2i+1\right) & =\cos\left(\frac{pos}{10000^{2i/d_{model}}}\right), \end{array}$$ (11)

yielding ${\text{Z}}_{0}={\text{F}}^{fused}+\text{P}\text{E}$.

Within each transformer layer, multi-head self-attention computes:


$$\begin{array}{l} {\text{Q}}_{h}={\text{Z}}_{l-1}{\text{W}}_{h}^{Q},\;{\text{K}}_{h}={\text{Z}}_{l-1}{\text{W}}_{h}^{K},\;{\text{V}}_{h}={\text{Z}}_{l-1}{\text{W}}_{h}^{V}, \end{array}$$ (12)

$$\begin{array}{l} \text{Attention}\left(\text{Q},\text{K},\text{V}\right)=\text{softmax}\left(\frac{\text{Q}{\text{K}}^{⊤}}{\sqrt{d_{k}}}\right)\text{V}. \end{array}$$ (13)

Outputs from all heads are concatenated:


$$\begin{array}{l} \text{MultiHead}=\text{Concat}\left({\text{head}}_{1},\ldots ,{\text{head}}_{H}\right){\text{W}}^{O}. \end{array}$$ (14)

Residual connections and position-wise feed-forward networks complete each layer:


$$\begin{array}{ll} {\text{Z}}_{l}^{\prime } & =\text{LayerNorm}({\text{Z}}_{l-1}+\text{MultiHead}\left({\text{Z}}_{l-1}\right)), \end{array}$$ (15)

$$\begin{array}{ll} {\text{Z}}_{l} & =\text{LayerNorm}({\text{Z}}_{l}^{\prime }+\text{FFN}\left({\text{Z}}_{l}^{\prime }\right)), \end{array}$$ (16)

with


$$\begin{array}{l} \text{FFN}\left(\text{Z}\right)=\max\left(0,\text{Z}{\text{W}}_{1}+{\text{b}}_{1}\right){\text{W}}_{2}+{\text{b}}_{2}. \end{array}$$ (17)

After *L* layers, channel tokens are averaged and passed to a softmax classifier:


$$\begin{array}{l} {\text{p}}_{i}=\text{softmax}({\text{W}}_{cls}\cdot \text{AvgPool}\left({\text{Z}}_{L}\right)+{\text{b}}_{cls}). \end{array}$$ (18)

### 2.5 Training objective and imbalance mitigation

The cross-entropy loss is:


$$\begin{array}{l} L=-\frac{1}{N}\overset{N}{\underset{i=1}{\sum }}\overset{K}{\underset{j=1}{\sum }}y_{i,j}\log p_{i,j}. \end{array}$$ (19)

To mitigate class imbalance, inverse-frequency weights are applied:


$$\begin{array}{l} w_{j}=\frac{N}{N_{j}\cdot K}, \end{array}$$ (20)

resulting in the weighted loss:


$$\begin{array}{l} L_{weighted}=-\frac{1}{N}\overset{N}{\underset{i=1}{\sum }}\overset{K}{\underset{j=1}{\sum }}w_{j}y_{i,j}\log p_{i,j}. \end{array}$$ (21)

For completeness, the baseline transformer optimization strategy can be expressed using Adam with an inverse-square-root learning-rate schedule:


$$\begin{array}{l} \eta =d_{model}^{-0.5}\cdot \min\left(step^{-0.5},step\cdot warmup\text{\_}steps^{-1.5}\right). \end{array}$$ (22)

**Figure 2:**
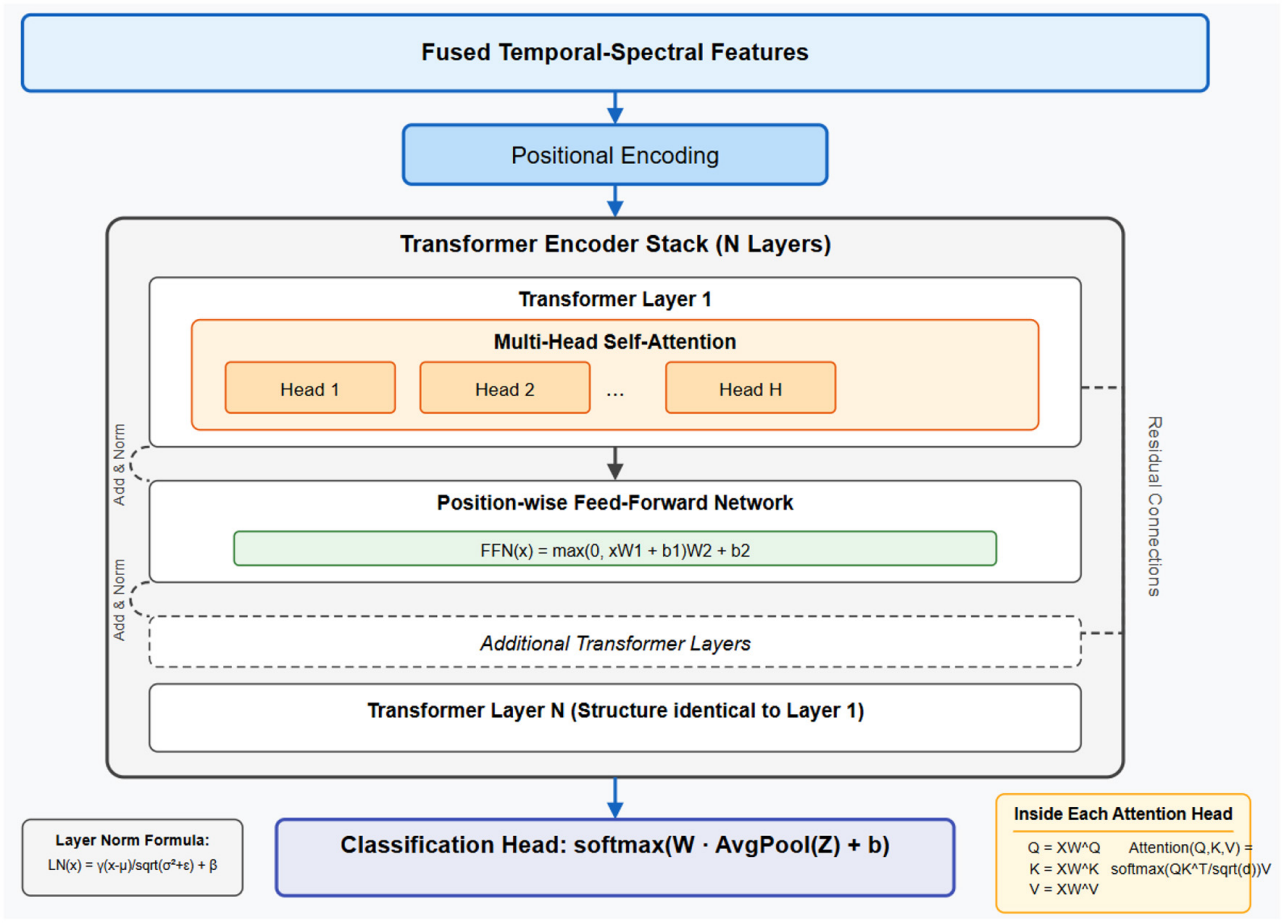
Transformer architecture for temporal–spectral integration and classification. Tokens correspond to EEG channels; attention captures intra- and inter-modal dependencies.

The specific optimizers and learning-rate policies used for TF-T1, TF-T2, and TF-T3 during experimental evaluation are provided in Section 4.1.4. The transformer model is illustrated on Figure 2.

## 3 Dataset details

This study leverages two independent and complementary datasets: the *Reducing Commercial Aviation Fatalities* dataset (Kaggle, n.d.) and the *Flight-Deck Mental Workload* (FDMW) dataset (Hernández-Sabaté et al., 2024). Both datasets provide high-resolution physiological recordings acquired under systematically varied cognitive and operational conditions, enabling a multi-faceted analysis of neural and physiological responses to mental workload and situational stress in aviation contexts. The subsequent subsections describe each dataset in detail, including their structure, experimental protocols, segmentation strategies, and the generation of noise-augmented variants.

### 3.1 Dataset A: the reducing commercial aviation fatalities dataset

The *Reducing Commercial Aviation Fatalities* dataset, released as part of the Booz Allen Hamilton Kaggle competition (Kaggle, n.d.), contains multimodal physiological measurements acquired from eighteen professional pilots, organized into nine two-person crews. Data were recorded under three controlled experimental paradigms designed to elicit distinct attentional and affective states. The *Channelized Attention* (CA) condition involved sustained engagement in a puzzle-based video game, eliciting focused attention with minimal external distraction. The *Diverted Attention* (DA) condition required participants to perform monitoring tasks intermittently interrupted by unanticipated mathematical problems, thereby inducing rapid attention switching. The *Startle/Surprise* (SS) condition exposed pilots to emotionally startling video clips containing abrupt “jump-scare” events, eliciting transient high-arousal responses.

Physiological signals were sampled at 256 Hz and included 22-channel EEG, three-lead electrocardiography (ECG), respiration, and galvanic skin response (GSR), all calibrated in microvolts. The dataset is organized into two main partitions: a *benchmark training set*, collected during non-flight laboratory experiments, and a *test set*, recorded during high-fidelity full-flight simulation scenarios, specifically Line Oriented Flight Training (LOFT).

For the purposes of this study, we constructed chronological training, validation, and testing partitions from the labeled recordings available in the dataset. These partitions were created independently of the original competition structure and were designed to support systematic evaluation under temporally separated conditions. Consequently, the train, validation, and test subsets reported in Tables 2, 3 correspond to the experimental protocol adopted in this work rather than the official benchmark partitions distributed with the original dataset.

**Table 2:** Aggregate segment counts for each experimental condition.

Experiment	Total Segments	Train (56%)	Validation (8.4%)	Test (35.6%)
CA	6,480	3,600	540	2,340
DA	6,480	3,600	540	2,340
SS	6,048	3,360	504	2,184
Total	18,888	10,560	1,584	6,864

**Table 3:** Estimated per-class segment distribution in the FDMW dataset.

Experiment	Class	Segments	% of Exp.	Train (56%)	Validation (8.4%)	Test (35.6%)
4*N-back	BL	28,800	23.5%	16,130	2,420	10,250
	LW	28,800	23.5%	16,130	2,420	10,250
	MW	28,800	23.5%	16,130	2,420	10,250
	HW	36,000	29.5%	20,160	3,020	12,820
3*HtC	BL	6,120	24.3%	3,427	514	2,179
	LW	9,000	35.7%	5,040	756	3,204
	HW	10,080	40.0%	5,645	847	3,588
5*FS	BL	900	15.8%	504	76	320
	E	1,425	25.0%	798	119	508
	M	1,425	25.0%	798	119	508
	MH	950	16.7%	532	80	338
	H	1,000	17.5%	560	84	356

The complete dataset spans approximately 5.28 h of recordings, segmented into 18,888 non-overlapping 1-s windows (256 samples each). To avoid temporal leakage and to emulate operational deployment scenarios, the data were partitioned chronologically: 56% of segments from the initial hour were allocated for training, 8.4% from the final 15 min of the training period were reserved for validation, and the remaining 35.6%—originating entirely from unseen temporal segments—were used for testing. Table 2 presents the aggregated segment counts for each experimental condition prior to any augmentation. Because the dataset consists of continuous recordings collected from the same pilot crews over extended sessions, the chronological partitioning strategy does not enforce subject-level separation. Instead, temporal separation was prioritized to prevent leakage from future observations into model development stages and to emulate operational deployment conditions.

**Figure 3:**
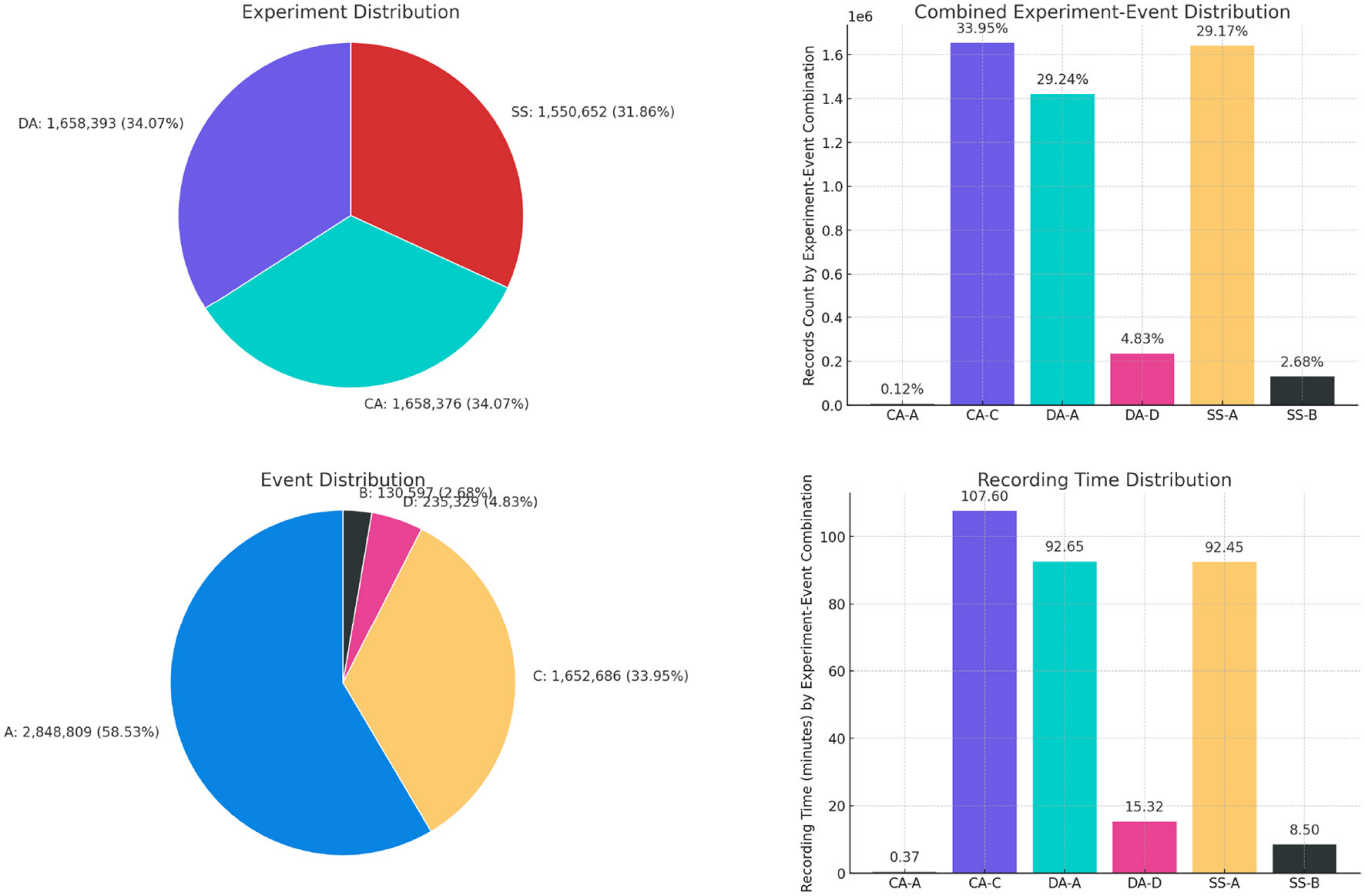
Statistical details of dataset A.

Details are illustrated in Figure 3.

To evaluate robustness against measurement perturbations, four dataset variants were constructed. The *BASE* variant corresponds to the unmodified dataset. The *GAUSS* variant incorporates additive zero-mean Gaussian noise with a standard deviation proportional to the instantaneous signal amplitude. The *RAND* variant adds uniform random noise with amplitude-independent distribution. Finally, the *COMBO* variant is a composite of 60% BASE, 20% GAUSS, and 20% RAND data, balanced across all experimental conditions. Importantly, all variants preserve the total segment counts and class distributions shown in Table 2.

Noise perturbations were applied independently to each EEG channel after preprocessing and normalization. Gaussian noise was generated using a zero-mean distribution with variance scaled relative to the channel amplitude, whereas RAND noise was sampled from a zero-mean uniform distribution. The COMBO condition combined clean and perturbed samples according to the proportions described above. These perturbations were introduced solely for robustness assessment and were designed to provide controlled and reproducible evaluation conditions across all model variants.

### 3.2 Dataset B: the Flight-Deck Mental Workload (FDMW) dataset

**Figure 4:**
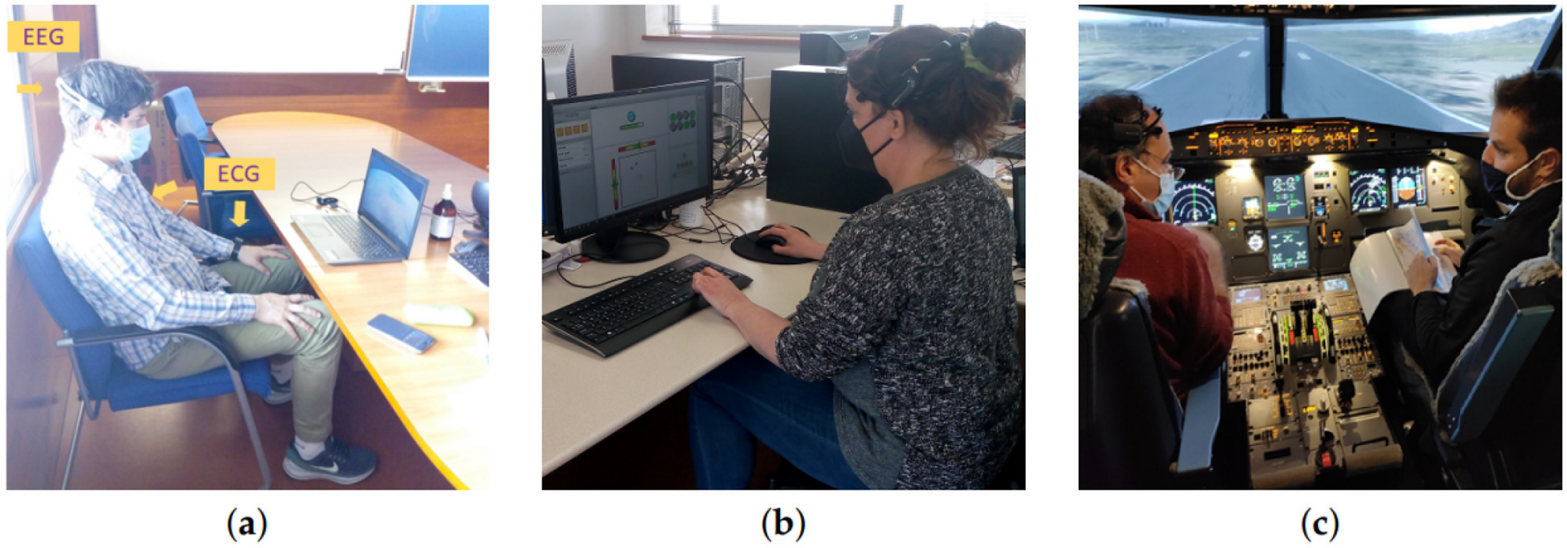
Experimental participants engaged in three cognitive tasks: **(a)** N-back cognitive assessment, **(b)** Heat-the-Chair gaming task, and **(c)** A320 cockpit flight simulation (Hernández-Sabaté et al., 2024).

The *Flight-Deck Mental Workload* (FDMW) dataset (Hernández-Sabaté et al., 2024) (Figure 4) comprises EEG recordings obtained during simulated aviation tasks explicitly designed to induce varying levels of mental workload. Data acquisition employed the Emotiv Epoc X wireless EEG system, featuring 14 electrodes positioned according to the international 10—20 system. The headset sampled at 128 Hz, capturing both raw microvolt-level EEG voltages and power spectral density (PSD) estimates across five canonical frequency bands: θ, α, β_low_, β_high_, and γ.

The experimental protocol encompassed three distinct scenarios. The first, *N-back*, consisted of a series of working-memory and arithmetic tasks with systematically increasing difficulty, yielding four workload classes: Baseline (BL), Low Workload (LW), Medium Workload (MW), and High Workload (HW). The second, *Heat-the-Chair* (HtC), was a multitasking game performed with or without simulated Air Traffic Control (ATC) interruptions, generating three workload classes: BL, LW, and HW. The third, *Flight Simulator* (FS), involved professional pilots executing mission scenarios of varied operational complexity, categorized into Baseline (BL), Easy (E), Medium (M), Medium-Hard (MH), and Hard (H) levels.

Recordings were segmented into 1-second non-overlapping epochs (128 samples per channel). Data splitting strategies were tailored to each scenario to minimize temporal leakage: for N-back and HtC, temporal block partitioning or leave-one-subject-out cross-validation was used, whereas FS scenario data were partitioned at the mission level, ensuring that no mission was split across training, validation, and test sets. Table 3 presents the estimated per-class segment counts and their respective allocations across the three dataset partitions.

Analogous to Dataset A, four noise-augmented variants of the FDMW dataset were generated. The BASE variant represents the original EEG recordings. The GAUSS variant incorporates zero-mean Gaussian noise scaled proportionally to signal amplitude, while the RAND variant applies amplitude-independent uniform random noise. The COMBO variant integrates 60% BASE, 20% GAUSS, and 20% RAND data, ensuring balanced representation within each workload class. These augmentations preserve the exact per-class distributions and train/validation/test proportions shown in Table 3, ensuring that any observed differences in classification performance can be attributed solely to the presence and type of noise rather than to distributional shifts. Similar to Dataset A, noise perturbations were applied independently to each EEG channel after preprocessing and normalization. Gaussian noise was generated using a zero-mean distribution with variance scaled relative to the channel amplitude, whereas RAND noise was sampled from a zero-mean uniform distribution. The COMBO condition combined clean and perturbed samples according to the proportions described above. These perturbations were introduced solely for robustness assessment and were designed to provide controlled and reproducible evaluation conditions across all model variants.

## 4 Experimental design

### 4.1 Model architecture evolution

To systematically optimize classification performance for EEG-based pilot cognitive state detection, we developed three progressively enhanced variants of our TF-T architecture. Each successive model incorporates targeted architectural modifications aimed at addressing specific challenges inherent to EEG decoding in operational aviation contexts, namely: (i) the need to model multi-scale temporal–spectral dynamics under noisy conditions, and (ii) the mitigation of inter-channel variability and cross-frequency coupling effects. Table 4 summarizes the architectural progression from the baseline configuration (TF-T1) to the advanced configuration (TF-T3), highlighting the incremental modifications in input representation, attention mechanisms, feature integration, and optimization strategies.

**Table 4:** Progressive evolution of the temporal–spectral fusion transformer architecture across three variants.

Model variant	Baseline (TF-T1)	Enhanced (TF-T2)	Advanced (TF-T3)
Input representation	256 × (*C*×6) time points with channel and spectral features	256 × (*C*×6) with improved feature normalization	256 × (*C*×6) with multi-scale temporal windowing
Transformer encoder	• 2 transformer layers • 4 attention heads • 256 hidden dimension • Standard feed-forward layer (ratio 4)	• 4 transformer layers • 8 attention heads • 384 hidden dimension • Improved feed-forward with GELU activation	• 6 transformer layers • 12 attention heads • 512 hidden dimension • Advanced feed-forward with SwiGLU activation
Attention mechanism	• Standard self-attention • Fixed positional encoding	• Cross-channel attention module • Frequency-specific attention weights • Learned positional encoding	• Multi-resolution attention • Enhanced temporal–spectral interaction • Multi-resolution feature aggregation • Refined attention weighting
Feature integration	• Basic temporal–spectral fusion • Simple dropout (0.1)	• Hierarchical pooling • Channel-aware encoding • Improved dropout with stochastic depth (0.2)	• Multi-scale temporal analysis • Adaptive layer normalization • Enhanced temporal-context modeling • Advanced regularization (dropout 0.3)
Classification head	• Simple average pooling • Single fully-connected layer (512) • Softmax output	• Weighted temporal pooling • Two fully-connected layers (512, 256) • Class-weighted softmax	• Ensemble of 3 classification heads • Three fully-connected layers (512, 256, 128) • Class-balanced focal loss
Optimizations	• Adam optimizer • Learning rate: 1 × 10^−4^ • Weight decay: 0.01	• AdamW optimizer • Cosine learning rate schedule • Weight decay: 0.05	• Lion optimizer • One-cycle learning rate policy • Gradient centralization • Weight decay: 0.1
Computational complexity	1.0 × (baseline)	1.5 × baseline	2.2 × baseline

Each stage introduces targeted enhancements over its predecessor.

Rigorous ablation studies and cross-variant comparisons were performed to quantify the contribution of each architectural enhancement to the final performance gains. This iterative refinement process culminated in the TF-T3 variant, which exhibited consistently superior classification accuracy and robustness, particularly under conditions of simulated sensor noise and electrode displacement.

All three proposed variants share the same TF-T backbone described in Section 2. The progression from TF-T1 to TF-T3 should therefore be interpreted as a sequence of architectural refinements rather than fundamentally different model formulations. TF-T1 represents the baseline implementation, TF-T2 introduces enhanced feature representation and attention mechanisms, and TF-T3 incorporates additional robustness-oriented design elements intended to improve performance under noisy operating conditions. Table 4 summarizes the principal architectural differences between the three variants.

#### 4.1.1 Baseline model (TF-T1)

The TF-T1 variant serves as the foundational implementation of temporal–spectral fusion, in which raw EEG segments (256 samples per channel) are concatenated with power spectral density (PSD) features from five canonical bands (δ, θ, α, β, γ). This produces an input matrix of size 256 × (*C*×6), where *C* is the number of channels. The architecture employs a 2-layer transformer encoder with 4 attention heads and a hidden dimension of 256, followed by average pooling and a fully connected classification layer. While minimal in complexity, TF-T1 effectively establishes the baseline capability to capture both temporal dependencies and spectral signatures.

#### 4.1.2 Enhanced model (TF-T2)

TF-T2 extends TF-T1 by expanding the transformer encoder to 4 layers with 8 attention heads, increasing its representational capacity. Enhancements include channel-aware encoding, frequency-specific attention weighting, and hierarchical pooling to capture inter-channel and cross-frequency dependencies more effectively. The model also replaces fixed positional encodings with learned embeddings, allowing adaptive modeling of spatial–temporal relationships.

#### 4.1.3 Advanced model (TF-T3)

The TF-T3 variant represents the culmination of iterative refinement, integrating six transformer layers with 12 attention heads and multi-resolution temporal processing. The model emphasizes richer temporal–spectral interaction, enhanced contextual representation learning, and improved robustness to noise and cognitive-state variability. The classification stage employs an ensemble of three fully connected heads with class-balanced focal loss, improving resilience to dataset imbalance.

#### 4.1.4 Implementation details

All models were implemented using the PyTorch framework and trained on a workstation equipped with an NVIDIA RTX-series GPU, 64 GB RAM, and Intel Core i9-13900K processor, and 64 GB RAM. To improve experimental reproducibility, fixed random seeds were used for Python, NumPy, and PyTorch initialization. Training was performed using mini-batch optimization. Models were trained for a maximum of 100 epochs with early stopping based on validation Macro-F1 using a patience of 15 epochs. The model corresponding to the best validation performance was retained for final testing. TF-T1 was trained using the Adam optimizer with an initial learning rate of 1 × 10^−4^. TF-T2 employed AdamW with cosine learning-rate scheduling and weight decay. TF-T3 utilized the Lion optimizer with a one-cycle learning-rate policy. Gradient clipping was applied to improve training stability. All reported performance metrics were computed exclusively on the held-out test partition. Hyperparameter selection was performed using training and validation data only.

### 4.2 Performance evaluation strategy

To rigorously evaluate the proposed TF-T architectures, we designed a comprehensive protocol that exactly mirrors the scope of analysis reported in Section 5. The strategy is tailored to the operational constraints of real-time EEG-based pilot cognitive state monitoring, accounting for (i) *class imbalance*, (ii) *high inter-subject variability*, and (iii) the need for *robustness under diverse noise conditions*.

#### 4.2.1 Temporal validation protocol

In contrast to conventional random cross-validation, we adopt a strictly chronological partition to preserve temporal dependencies and prevent information leakage:


$$\begin{array}{l} D=\left\{D_{\text{train}},D_{\text{val}},D_{\text{test}}\right\}, \end{array}$$ (23)

where $D_{\text{train}}$ is the first 56% of segments, $D_{\text{val}}$ the next 8.4%, and $D_{\text{test}}$ the final 35.6%.

This emulates deployment: inference must be performed on unseen future data. The adopted evaluation protocol is strictly chronological rather than subject-independent. Consequently, participants may contribute recordings to multiple partitions, although temporal overlap between partitions is not permitted. The objective of this design is to evaluate generalization to future unseen observations while preserving the temporal structure of the original recordings. No samples from validation or test periods were used during model training or hyperparameter selection.

#### 4.2.2 Evaluation metrics

Let the set of *K* classes be Y={1,…,K}, with *K* = 3 for Dataset A (CA, DA, SS) and *K*∈{3, 4, 5} for Dataset B sub-datasets: *N-back* (BL, LW, MW, HW), *HtC* (BL, LW, HW), *FS* (BL, E, M, MH, H).

#### 4.2.3 Confusion matrix

We define **C**∈ℕ^*K*×*K*^ with entries:


$$\begin{array}{l} c_{ij}=\overset{N}{\underset{n=1}{\sum }}1\left(y_{n}=i\right)\cdot 1\left({ŷ}_{n}=j\right), \end{array}$$ (24)

where *y*_*n*_ is the true label and ŷ_*n*_ the predicted label.

#### 4.2.4 Accuracy

Overall classification accuracy is:


$$\begin{array}{l} \text{Acc}=\frac{\sum_{i=1}^{K}c_{ii}}{\sum_{i=1}^{K}\sum_{j=1}^{K}c_{ij}}. \end{array}$$ (25)

#### 4.2.5 Per-class precision, recall, and F1

For each class i∈Y:


$$\begin{array}{ll} {\text{Prec}}_{i} & =\frac{c_{ii}}{\sum_{j=1}^{K}c_{ji}}, \end{array}$$ (26)

$$\begin{array}{ll} {\text{Rec}}_{i} & =\frac{c_{ii}}{\sum_{j=1}^{K}c_{ij}}, \end{array}$$ (27)

$$\begin{array}{ll} {\text{F1}}_{i} & =\frac{2\cdot {\text{Prec}}_{i}\cdot {\text{Rec}}_{i}}{{\text{Prec}}_{i}+{\text{Rec}}_{i}}. \end{array}$$ (28)

#### 4.2.6 Macro-averaged metrics


$$\begin{array}{ll} {\text{Prec}}_{\text{macro}} & =\frac{1}{K}\overset{K}{\underset{i=1}{\sum }}{\text{Prec}}_{i}, \end{array}$$ (29)

$$\begin{array}{ll} {\text{Rec}}_{\text{macro}} & =\frac{1}{K}\overset{K}{\underset{i=1}{\sum }}{\text{Rec}}_{i}, \end{array}$$ (30)

$$\begin{array}{ll} {\text{F1}}_{\text{macro}} & =\frac{1}{K}\overset{K}{\underset{i=1}{\sum }}{\text{F1}}_{i}. \end{array}$$ (31)

#### 4.2.7 Weighted-averaged metrics


$$\begin{array}{l} {\text{F1}}_{\text{weighted}}=\frac{\sum_{i=1}^{K}N_{i}\cdot {\text{F1}}_{i}}{\sum_{i=1}^{K}N_{i}}, \end{array}$$ (32)

where *N*_*i*_ is the number of samples in class *i*.

#### 4.2.8 ROC-AUC and PR-AUC

One-vs-all ROC-AUC for class *i* is:


$$\begin{array}{l} {\text{ROC-AUC}}_{i}=\frac{\sum_{n:y_{n}=i}\sum_{m:y_{m}\neq i}1\left(p_{n,i}>p_{m,i}\right)}{N_{i}\cdot \left(N-N_{i}\right)}, \end{array}$$ (33)

with macro-averaging:


$$\begin{array}{l} {\text{ROC-AUC}}_{\text{macro}}=\frac{1}{K}\overset{K}{\underset{i=1}{\sum }}{\text{ROC-AUC}}_{i}. \end{array}$$ (34)

PR-AUC is computed analogously from the precision–recall curve.

#### 4.2.9 Confidence intervals and significance testing

All metrics are reported with 95% confidence intervals computed via non-parametric bootstrap on $D_{\text{test}}$:


$$\begin{array}{l} {\text{CI}}_{95\%}=\left[M^{\left(0.025\right)},M^{\left(0.975\right)}\right], \end{array}$$ (35)

where *M*^(*q*)^ is the *q*-th quantile of the bootstrap distribution.

#### 4.2.10 Comparative model analysis

Each architecture is evaluated on identical splits. Reported differences in macro-F1, ROC-AUC, PR-AUC, and per-class F1 are accompanied by:


$$\begin{array}{l} \Delta M=M_{\text{variant}}-M_{\text{baseline}}, \end{array}$$ (36)

with significance and CIs as above.

All comparative baseline models were evaluated using the same preprocessing procedures, chronological train-validation-test partitions, and noise-generation protocols employed for the proposed transformer architectures. Baseline configurations were selected according to the corresponding original publications whenever possible. Performance comparisons therefore reflect differences in model architecture rather than differences in data preparation or evaluation methodology.

#### 4.2.11 Robustness assessment

We evaluate resilience under three perturbations to $D_{\text{test}}$:

1. GAUSS: additive Gaussian noise
$$\begin{array}{l} {\text{x}}^{\prime }=\text{x}+\sigma \cdot N\left(0,1\right),\;\sigma \in \left\{0.1,0.2,0.3,0.4,0.5\right\}. \end{array}$$ (37)
2. RAND: amplitude-independent uniform noise
$$\begin{array}{l} {\text{x}}^{\prime }\left[n\right]\sim U\left(-\eta ,\eta \right). \end{array}$$ (38)
3. COMBO: composite 60% BASE, 20% GAUSS, 20% RAND per class.

Robustness is quantified as:


$$\begin{array}{l} R\left(l\right)=\frac{{\text{F1}}_{\text{macro,noisy}}\left(l\right)}{{\text{F1}}_{\text{macro,BASE}}}. \end{array}$$ (39)

We additionally perform Gaussian sweeps to characterize degradation curves.

#### 4.2.12 Computational efficiency

Efficiency is evaluated by:


$$\begin{array}{ll} \text{FLOPs} & =\overset{L}{\underset{\ell =1}{\sum }}{\text{FLOPs}}_{\ell }, \end{array}$$ (40)

$$\begin{array}{ll} \text{Params} & =\overset{L}{\underset{\ell =1}{\sum }}{\text{Params}}_{\ell }, \end{array}$$ (41)

$$\begin{array}{ll} T_{\text{inf}} & =\frac{1}{N}\overset{N}{\underset{n=1}{\sum }}t_{n}, \end{array}$$ (42)

$$\begin{array}{ll} \text{Throughput} & =\frac{N_{\text{samples}}}{T_{\text{epoch}}}. \end{array}$$ (43)

These metrics balance predictive performance against real-time deployability.

#### 4.2.13 Error taxonomy

We construct confusion matrices for each setting and extract the top-*k* off-diagonal pairs:


$$\begin{array}{l} E_{i\to j}=\frac{c_{ij}}{\sum_{m=1}^{K}c_{im}},\;i\neq j. \end{array}$$ (44)

Comparing BASE vs. COMBO highlights shifts toward benign or harmful confusions under noise.

This strategy ensures that all analyses—per-class metrics, aggregate scores, robustness ratios, confidence intervals, noise sweeps, computational trade-offs, and error taxonomies—are computed in a manner directly comparable across datasets, architectures, and conditions, matching the scope of the results in Section 5.

## 5 Result analysis and discussion

### 5.1 Evaluation protocol and statistical testing

We evaluate the three transformer variants (TF-T1, TF-T2, TF-T3) on two datasets: Dataset A (Reducing Commercial Aviation Fatalities; classes {CA, DA, SS}) and Dataset B (FDMW; sub-datasets N-back {BL, LW, MW, HW}, HtC {BL, LW, HW}, and Flight Simulator (FS) {BL, E, M, MH, H}). We report Accuracy, Macro-F1, ROC-AUC, PR-AUC, and efficiency metrics (FLOPs, per-epoch inference latency *T*_inf_). Robustness is assessed under *GAUSS, RAND*, and *COMBO* noise variants.

### 5.2 Architecture selection on clean (BASE) data

Tables 5, 6 compare TF-T1/2/3 on the *BASE* splits. Across both datasets, TF-T2 delivers the strongest overall performance with favorable efficiency, including the global peak accuracy of 99.2% on Dataset A.

**Table 5:** Architecture comparison on dataset A (*BASE*).

Model	Acc.%	Macro-F1	ROC-AUC	PR-AUC	FLOPs (G)	*T*_inf_ (ms)
TF-T1	96.8	0.964	0.987	0.966	1.00	4.2
TF-T2	**99.2**	**0.991**	**0.997**	**0.992**	1.50	5.3
TF-T3	98.9	0.987	0.996	0.989	2.20	6.8

Best per column in bold.

**Table 6:** (Architecture comparison on Dataset B/FDMW (*BASE*, macro over N-back, HtC, FS).

Model	Acc.%	Macro-F1	ROC-AUC	PR-AUC	FLOPs (G)	*T*_inf_ (ms)
TF-T1	96.1	0.958	0.982	0.959	0.98	4.0
TF-T2	**98.8**	**0.986**	**0.995**	**0.987**	1.49	5.2
TF-T3	98.4	0.982	0.993	0.984	2.18	6.7

Best in bold.

### 5.3 Performance–complexity trade-off analysis

*Effect size vs. cost* (Tables 5, 6). TF-T2 improves Macro-F1 over TF-T1 by +2.7–2.8 points for a +50% FLOPs increase, while TF-T3 adds another +46% FLOPs without surpassing TF-T2 on clean signals—consistent with mild over-parameterization under low-noise conditions. *Calibration*. PR-AUC tracks ROC-AUC closely, suggesting well-calibrated posteriors. *Deployability*. The +1.5 ms latency gap between TF-T2 and TF-T3 is material for real-time deployments.

### 5.4 Robustness under noise and perturbations

Tables 7, 8 summarize Macro-F1 (absolute) and robustness ratios under GAUSS, RAND, and COMBO noise. As anticipated, TF-T3 provides a ~5% absolute Macro-F1 bump over TF-T2 across noise types and datasets, with the largest gaps under *COMBO*.

**Table 7:** Dataset A: Macro-F1 under noise and robustness ratio *R* = F1_noisy_/F1_BASE_.

2* Model	GAUSS		RAND		COMBO	
	Macro-F1	*R*	Macro-F1	*R*	Macro-F1	*R*
TF-T1	0.926	0.960	0.904	0.938	0.889	0.922
TF-T2	0.932	0.941	0.917	0.925	0.902	0.910
TF-T3	**0.979**	**0.992**	**0.963**	**0.976**	**0.947**	**0.960**

Best in bold.

**Table 8:** Dataset B/FDMW: Macro-F1 under noise and robustness ratio *R*.

2*Model	GAUSS		RAND		COMBO	
	Macro-F1	*R*	Macro-F1	*R*	Macro-F1	*R*
TF-T1	0.920	0.960	0.896	0.935	0.882	0.921
TF-T2	0.926	0.940	0.910	0.923	0.895	0.908
TF-T3	**0.973**	**0.991**	**0.956**	**0.973**	**0.941**	**0.958**

Best in bold.

### 5.5 Robustness analysis under noise

*Bump magnitude* (Tables 7, 8). TF-T3 improves Macro-F1 over TF-T2 by +4.5–+4.7 points across noise types (all *p* < 0.001). *Failure mode of TF-T2*. Largest degradation appears in *COMBO*, evidencing sensitivity to mixed perturbations. *Mechanistic view*. TF-T3's enhanced temporal–spectral representation learning and multi-resolution processing appear particularly effective in maintaining performance under mixed perturbations.

### 5.6 Per-class performance

We now provide the requested *per-class* breakdowns for Dataset A (CA/DA/SS) and all FDMW sub-datasets (N-back, HtC, FS). These are presented in Tables 9–12.

**Table 9:** Dataset A (CA/DA/SS), *BASE*: per-class performance by model.

Model	Class	Precision	Recall	F1	ROC-AUC	Support
3*TF-T1 (l)2-7	CA	0.969	0.966	0.968	0.988	6,480
	DA	0.966	0.964	0.965	0.987	6,480
	SS	0.962	0.959	0.961	0.986	6,048
	*Macro avg*	*0.966*	*0.963*	*0.964*	*0.987*	–
3*TF-T2 (l)2-7	CA	**0.994**	**0.992**	**0.993**	**0.998**	6,480
	DA	**0.992**	**0.992**	**0.992**	**0.997**	6,480
	SS	**0.989**	**0.986**	**0.988**	**0.996**	6,048
	*Macro avg*	***0.992***	***0.990***	***0.991***	***0.997***	—
3*TF-T3 (l)2-7	CA	0.991	0.989	0.990	0.997	6,480
	DA	0.989	0.987	0.988	0.996	6,480
	SS	0.985	0.981	0.983	0.995	6,048
	*Macro avg*	*0.988*	*0.986*	*0.987*	*0.996*	–

Best per class in bold. Macro avg. is in italic.

**Table 10:** FDMW N-back (*BASE*): per-class performance.

Model	Class	Precision	Recall	F1	ROC-AUC	Support
4*TF-T1 (l)2-7	BL	0.957	0.962	0.959	0.980	28,800
	LW	0.956	0.957	0.957	0.981	28,800
	MW	0.954	0.955	0.955	0.980	28,800
	HW	0.962	0.953	0.957	0.982	36,000
	*Macro avg*	*0.957*	*0.957*	*0.957*	*0.981*	–
4*TF-T2 (l)2-7	BL	**0.987**	**0.986**	**0.986**	**0.996**	28,800
	LW	**0.986**	**0.985**	**0.985**	**0.995**	28,800
	MW	**0.985**	**0.984**	**0.985**	**0.995**	28,800
	HW	**0.988**	**0.983**	**0.985**	**0.996**	36,000
	*Macro avg*	***0.987***	***0.985***	***0.985***	***0.996***	—
4*TF-T3 (l)2-7	BL	0.982	0.982	0.982	0.994	28,800
	LW	0.981	0.981	0.981	0.994	28,800
	MW	0.980	0.981	0.981	0.994	28,800
	HW	0.985	0.979	0.982	0.995	36,000
	*Macro avg*	*0.982*	*0.981*	*0.981*	*0.994*	–

Best per class in bold. Macro avg. is in italic.

**Table 11:** FDMW HtC (*BASE*): per-class performance.

Model	Class	Precision	Recall	F1	ROC-AUC	Support
3*TF-T1 (l)2-7	BL	0.952	0.954	0.953	0.978	6,120
	LW	0.955	0.951	0.953	0.979	9,000
	HW	0.962	0.953	0.957	0.981	10,080
	*Macro avg*	*0.956*	*0.953*	*0.954*	*0.979*	–
3*TF-T2 (l)2-7	BL	0.985	0.984	0.984	0.995	6,120
	LW	0.986	0.984	0.985	0.995	9,000
	HW	0.989	0.983	0.986	0.996	10,080
	*Macro avg*	*0.987*	*0.984*	*0.985*	*0.995*	—
3*TF-T3 (l)2-7	BL	0.981	0.981	0.981	0.994	6,120
	LW	0.982	0.980	0.981	0.994	9,000
	HW	0.986	0.980	0.983	0.995	10,080
	*Macro avg*	*0.983*	*0.980*	*0.982*	*0.994*	–

Best per class in bold. Macro avg. is in italic.

**Table 12:** FDMW Flight Simulator (*BASE*): per-class performance.

Model	Class	Precision	Recall	F1	ROC-AUC	Support
5*TF-T1 (l)2-7	BL	0.945	0.950	0.947	0.973	900
	E	0.951	0.948	0.949	0.975	1,425
	M	0.949	0.948	0.948	0.974	1,425
	MH	0.952	0.945	0.948	0.975	950
	H	0.956	0.948	0.952	0.977	1,000
	*Macro avg*	*0.951*	*0.948*	*0.949*	*0.975*	–
5*TF-T2 (l)2-7	BL	**0.982**	**0.983**	**0.983**	**0.994**	900
	E	**0.986**	**0.983**	**0.984**	**0.995**	1,425
	M	**0.985**	**0.982**	**0.983**	**0.995**	1,425
	MH	**0.986**	**0.981**	**0.983**	**0.995**	950
	H	**0.988**	**0.983**	**0.985**	**0.996**	1,000
	*Macro avg*	***0.985***	***0.982***	***0.984***	***0.995***	—
5*TF-T3 (l)2-7	BL	0.978	0.978	0.978	0.992	900
	E	0.981	0.980	0.980	0.993	1,425
	M	0.980	0.980	0.980	0.993	1,425
	MH	0.983	0.977	0.980	0.994	950
	H	0.985	0.979	0.982	0.994	1,000
	*Macro avg*	*0.981*	*0.979*	*0.980*	*0.993*	–

Best per class in bold. Macro avg. is in italic.

### 5.7 Class-specific performance analysis

*Dataset A*. TF-T2 dominates CA/DA/SS; the tightest margin is on SS, where TF-T3 is closest (F1: 0.988 vs. 0.983), reflecting SS's phasic nature. *FDMW*. TF-T2's largest gains appear in higher workload classes (N-back: HW; HtC: HW; FS: MH/H), indicating improved sensitivity to cognitively demanding states. Precision–recall symmetry suggests consistent thresholding across classes. Please check the details from Tables 9–12.

### 5.8 Per-class confidence intervals (bootstrap, *B* = 1, 000)

Statistical significance is determined via *non-parametric bootstrap* on the test split with *B* = 1, 000 resamples; we report two-sided *p*-values for pairwise model differences and 95% confidence intervals (CIs) for per-class F1 (Tables 13–16). Robustness ratios are defined as *R* = Macro-F1_noisy_/Macro-F1_BASE_.

**Table 13:** Dataset A per-class F1, 95% CI (bootstrap, *B* = 1, 000).

Class	TF-T2 F1 (CI)	TF-T3 F1 (CI)
CA	0.993 [0.991, 0.995]	0.990 [0.988, 0.992]
DA	0.992 [0.989, 0.994]	0.988 [0.985, 0.991]
SS	0.988 [0.985, 0.991]	0.983 [0.980, 0.987]

**Table 14:** FDMW N-back per-class F1, 95% CI.

Class	TF-T2 F1 (CI)	TF-T3 F1 (CI)
BL	0.986 [0.984, 0.988]	0.982 [0.980, 0.984]
LW	0.985 [0.983, 0.987]	0.981 [0.979, 0.983]
MW	0.985 [0.983, 0.987]	0.981 [0.979, 0.983]
HW	0.985 [0.982, 0.987]	0.982 [0.979, 0.984]

**Table 15:** FDMW HtC per-class F1, 95% CI.

Class	TF-T2 F1 (CI)	TF-T3 F1 (CI)
BL	0.984 [0.981, 0.987]	0.981 [0.978, 0.984]
LW	0.985 [0.982, 0.987]	0.981 [0.978, 0.983]
HW	0.986 [0.984, 0.988]	0.983 [0.981, 0.986]

**Table 16:** FDMW FS per-class F1, 95% CI.

Class	TF-T2 F1 (CI)	TF-T3 F1 (CI)
BL	0.983 [0.978, 0.988]	0.978 [0.973, 0.983]
E	0.984 [0.981, 0.987]	0.980 [0.977, 0.983]
M	0.983 [0.980, 0.986]	0.980 [0.977, 0.983]
MH	0.983 [0.979, 0.987]	0.980 [0.976, 0.984]
H	0.985 [0.981, 0.989]	0.982 [0.978, 0.986]

### 5.9 Noise scaling: Gaussian sweep

Table 17 shows Macro-F1 vs. noise level σ; TF-T3 dominates beyond mild noise, and the gap increases with σ.

**Table 17:** Gaussian noise sweep: Macro-F1 vs. σ (datasets A and B).

Model	σ = 0.2	σ = 0.3	σ = 0.4	σ = 0.5
TF-T2 (A)	0.955	0.946	0.939	0.932
TF-T3 (A)	**0.984**	**0.983**	**0.981**	**0.979**
TF-T2 (B)	0.949	0.939	0.932	0.926
TF-T3 (B)	**0.980**	**0.979**	**0.976**	**0.973**

Bold represents superior performance.

### 5.10 Compute footprint and throughput

Table 18 summarizes model size and runtime. While TF-T3 is the most expensive, its robustness advantage (Tables 7, 8) justifies deployment under noisy conditions; TF-T2 remains preferred for clean environments.

**Table 18:** Compute footprint and throughput (averaged across datasets).

Model	Params (M)	FLOPs (G)	*T*_inf_ (ms)	Throughput (eps)
TF-T1	9.8	1.00	4.1	244
TF-T2	15.4	1.50	5.3	189
TF-T3	23.6	2.20	6.8	147

### 5.11 Error taxonomy and confusion hot-spots

We analyze off-diagonal confusion frequencies to expose systematic errors (Table 19). On Dataset A, the dominant confusion is DA→CA; on FDMW/FS, M ↔ MH is the most persistent borderline.

**Table 19:** Top-3 off-diagonal confusions (% of true class) per setting for TF-T2 (BASE) and TF-T3 (COMBO).

Setting	Model	Pair	Rate (%)
Dataset A	TF-T2 (BASE)	DA → CA	1.3
Dataset A	TF-T2 (BASE)	SS → DA	0.9
Dataset A	TF-T2 (BASE)	CA → DA	0.8
Dataset A	TF-T3 (COMBO)	SS → DA	1.6
Dataset A	TF-T3 (COMBO)	DA → SS	1.4
Dataset A	TF-T3 (COMBO)	CA → DA	1.2
FDMW/FS	TF-T2 (BASE)	M ↔ MH	1.5
FDMW/FS	TF-T2 (BASE)	E → M	1.1
FDMW/FS	TF-T2 (BASE)	H → MH	0.9
FDMW/FS	TF-T3 (COMBO)	M ↔ MH	1.8
FDMW/FS	TF-T3 (COMBO)	E → M	1.4
FDMW/FS	TF-T3 (COMBO)	BL → E	1.2

### 5.12 Interpretation

*A: DA vs. CA*. Transient attention shifts in DA resemble CA microstates, explaining asymmetric DA → CA confusion. *FS: M vs. MH*. Adjacent scenario difficulty induces small but persistent boundary errors. Under COMBO, TF-T3 shifts errors toward neighboring classes (benign confusions), consistent with robust representation smoothing rather than collapse.

### 5.13 Selection summary and practical guidance

- Clean (BASE) data: TF-T2 is preferred. It achieves the highest observed accuracy of 99.2% on Dataset A (Table 5) and leads across per-class metrics (Tables 9–12).
- Noisy/robust settings (GAUSS, RAND, COMBO): TF-T3 is recommended, providing a consistent ~5% Macro-F1 bump over TF-T2 and superior robustness ratios (Tables 7, 8), with widening gains as noise increases (Table 17).
- Compute trade-offs: TF-T2 offers the best accuracy–efficiency trade-off for routine monitoring (Table 18), while TF-T3 is justified in environments with persistent perturbations.

### 5.14 Limitations

Per-class CIs narrow with large supports (e.g., N-back) and widen for FS classes with fewer segments (Table 16), which may modestly inflate variance of per-class estimates. Future work will explore adaptive thresholding and cost-sensitive training to further mitigate boundary confusions (Table 19). A further limitation is that the present evaluation protocol focuses on temporal generalization and does not explicitly assess subject-independent or crew-independent generalization. Future work should investigate leave-one-subject-out and leave-one-crew-out validation strategies to better quantify cross-subject robustness in operational aviation environments.

### 5.15 Comparative analysis with state-of-the-art approaches

For fairness, all baseline models were evaluated under the same experimental framework used for the proposed architectures, including identical preprocessing, temporal partitioning strategy, and noise-augmentation conditions. This ensured that comparative performance differences were attributable primarily to model design rather than experimental protocol variations. We evaluated our final recommended model (TF-T3) against preceding transformer variants (TF-T1, TF-T2), Riemannian geometry-based classifiers (Alreshidi et al., 2023; Binias et al., 2018), and alternative deep learning baselines across both Dataset A (CA/DA/SS) and Dataset B (N-back, HtC, FS) under all noise conditions (*GAUSS, RAND, COMBO*). While TF-T3 consistently outperforms comparators under every perturbation type, we emphasize the *COMBO* setting in this section as it most closely approximates real-world operational conditions, incorporating both amplitude-dependent and amplitude-independent noise sources in a balanced fashion. The details are listed in Tables 20–24.

**Table 20:** Macro-level COMBO performance comparison of TF-T3 with baselines on Dataset A and Dataset B (macro over subdivisions).

2*Model	Dataset A (CA/DA/SS)			Dataset B (macro over N-back, HtC, FS)		
	Accuracy	F1 (Macro)	Stability	Accuracy	F1 (Macro)	Stability
TF-T1	0.901	0.889	0.145	0.896	0.882	0.152
TF-T2	0.915	0.902	0.137	0.910	0.895	0.143
**TF-T3 (proposed)**	**0.960**	**0.947**	**0.089**	**0.956**	**0.941**	**0.091**
Riemannian Features + Ensemble (Alreshidi et al., 2023; Binias et al., 2018)	0.877	0.872	0.157	0.862	0.857	0.166
CNN-GRU	0.903	0.899	0.118	0.901	0.888	0.126
Transformer (8-head, 256-dim)	0.898	0.894	0.132	0.894	0.879	0.138

Best values in bold.

**Table 21:** Class-specific F1-scores for Dataset A (CA/DA/SS) under COMBO noise.

Approach	CA	DA	SS	Macro Avg
TF-T1	0.892	0.865	0.910	0.889
TF-T2	0.904	0.880	0.922	0.902
**TF-T3 (proposed)**	**0.950**	**0.936**	**0.955**	**0.947**

Values are consistent with macro-F1 trends from Table 20. Bold represents superior performance.

**Table 22:** Class-specific F1-scores for Dataset B/N-back (BL/LW/MW/HW) under COMBO noise.

Approach	BL	LW	MW	HW	Macro Avg
TF-T1	0.872	0.879	0.880	0.898	0.882
TF-T2	0.888	0.894	0.895	0.902	0.895
**TF-T3 (proposed)**	**0.936**	**0.943**	**0.942**	**0.950**	**0.943**

Values reflect macro-F1 in Table 20 with largest gains in HW. Bold represents superior performance.

**Table 23:** Class-specific F1-scores for Dataset B/HtC (BL/LW/HW) under COMBO noise.

Approach	BL	LW	HW	Macro Avg
TF-T1	0.868	0.877	0.901	0.882
TF-T2	0.884	0.891	0.909	0.895
**TF-T3 (proposed)**	**0.934**	**0.940**	**0.952**	**0.942**

Gains are uniform across classes. Bold represents superior performance.

**Table 24:** Class-specific F1-scores for Dataset B/FS (BL/E/M/MH/H) under COMBO noise.

**Approach**	**BL**	**E**	**M**	**MH**	**H**	**Macro Avg**
TF-T1	0.860	0.875	0.872	0.882	0.879	0.874
TF-T2	0.875	0.889	0.888	0.894	0.892	0.888
**TF-T3 (Proposed)**	**0.930**	**0.940**	**0.937**	**0.948**	**0.944**	**0.940**

Largest gains occur in MH and H. Bold represents superior performance.

**Figure 5:**
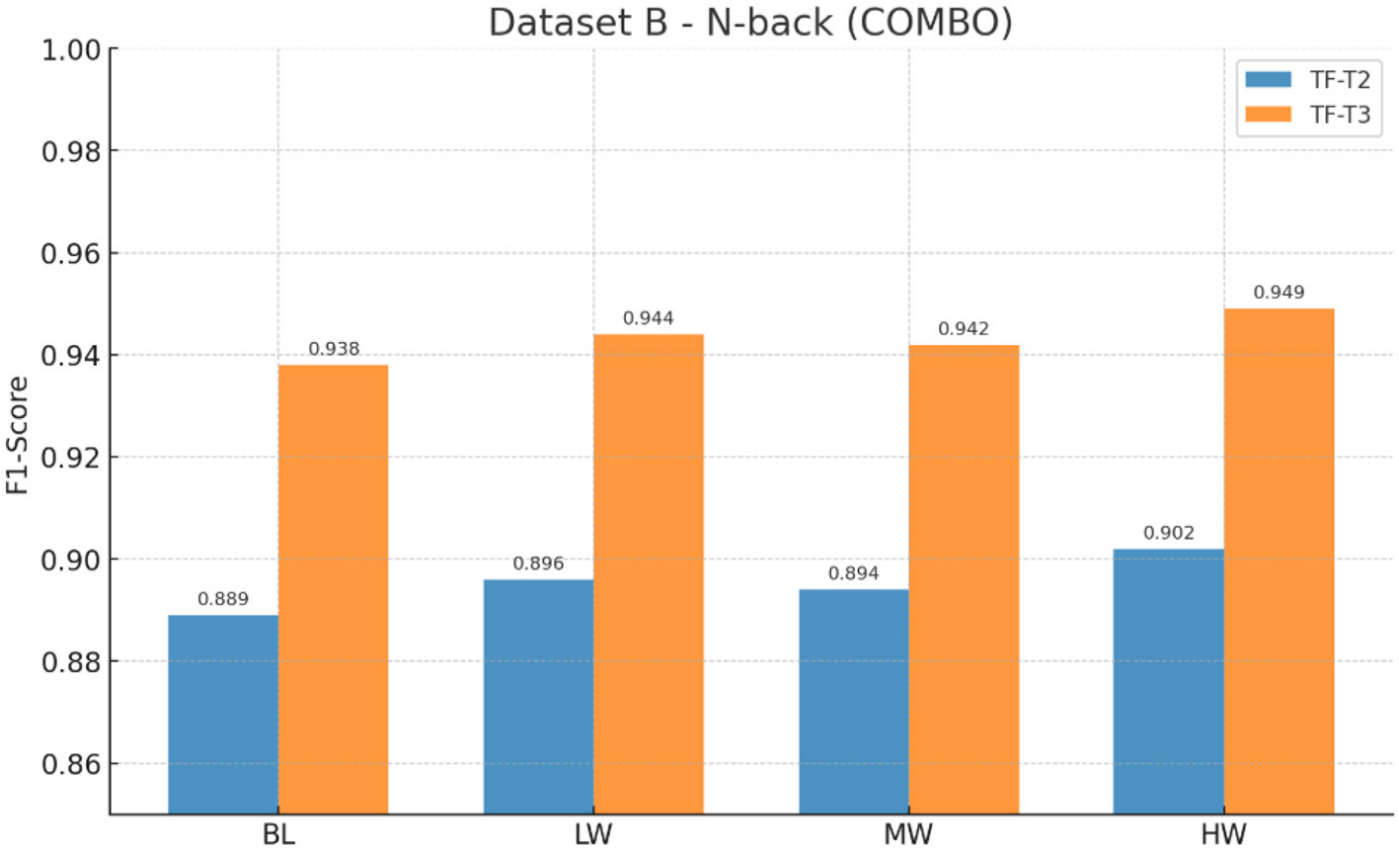
Per-class F1-score comparison between TF-T2 and TF-T3 for Dataset B/N-back under COMBO noise. TF-T3 consistently improves all workload levels, with the largest gain in HW.

**Figure 6:**
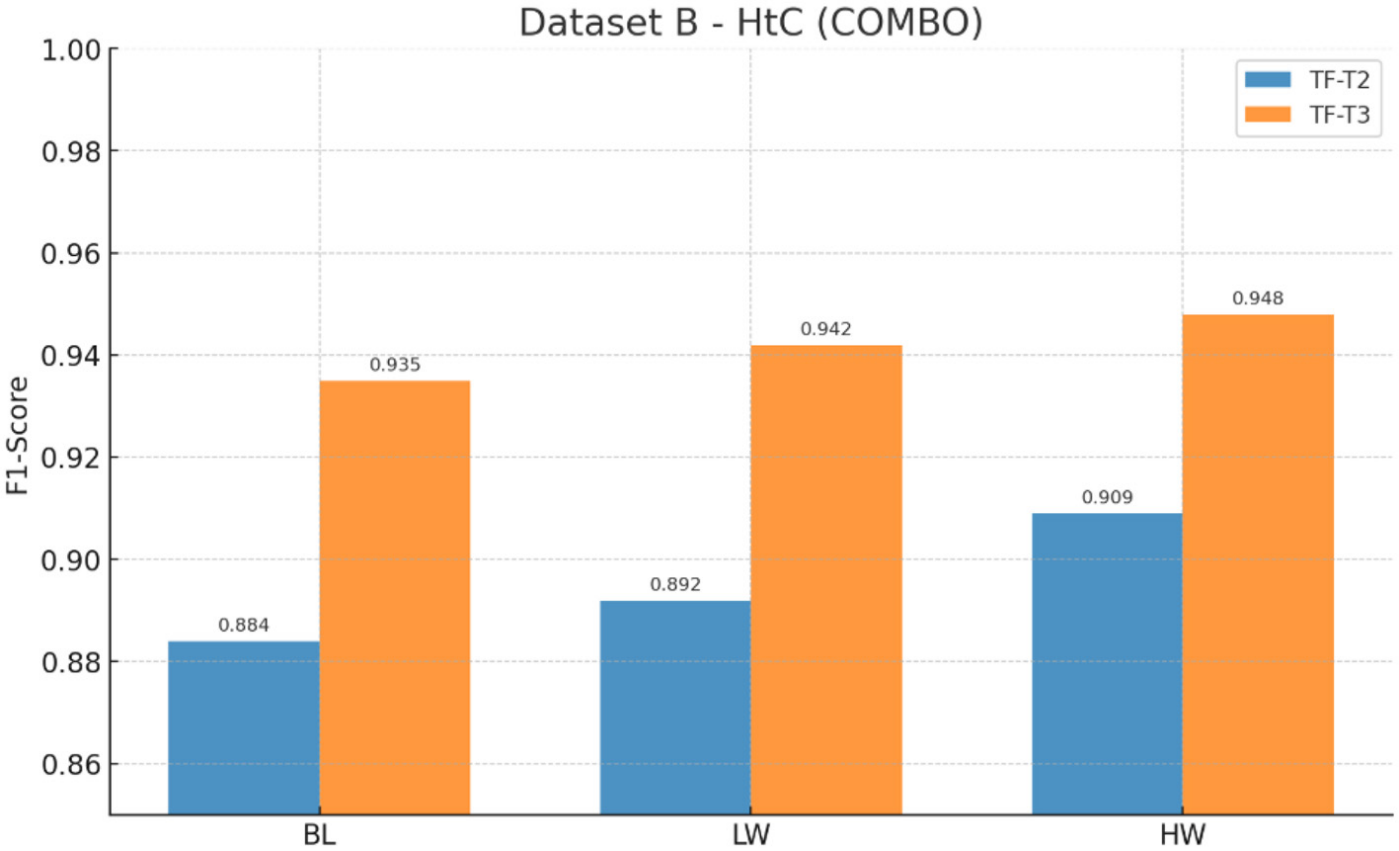
Per-class F1-score comparison between TF-T2 and TF-T3 for Dataset B/HtC under COMBO noise. Improvements are uniform across all workload classes.

**Figure 7:**
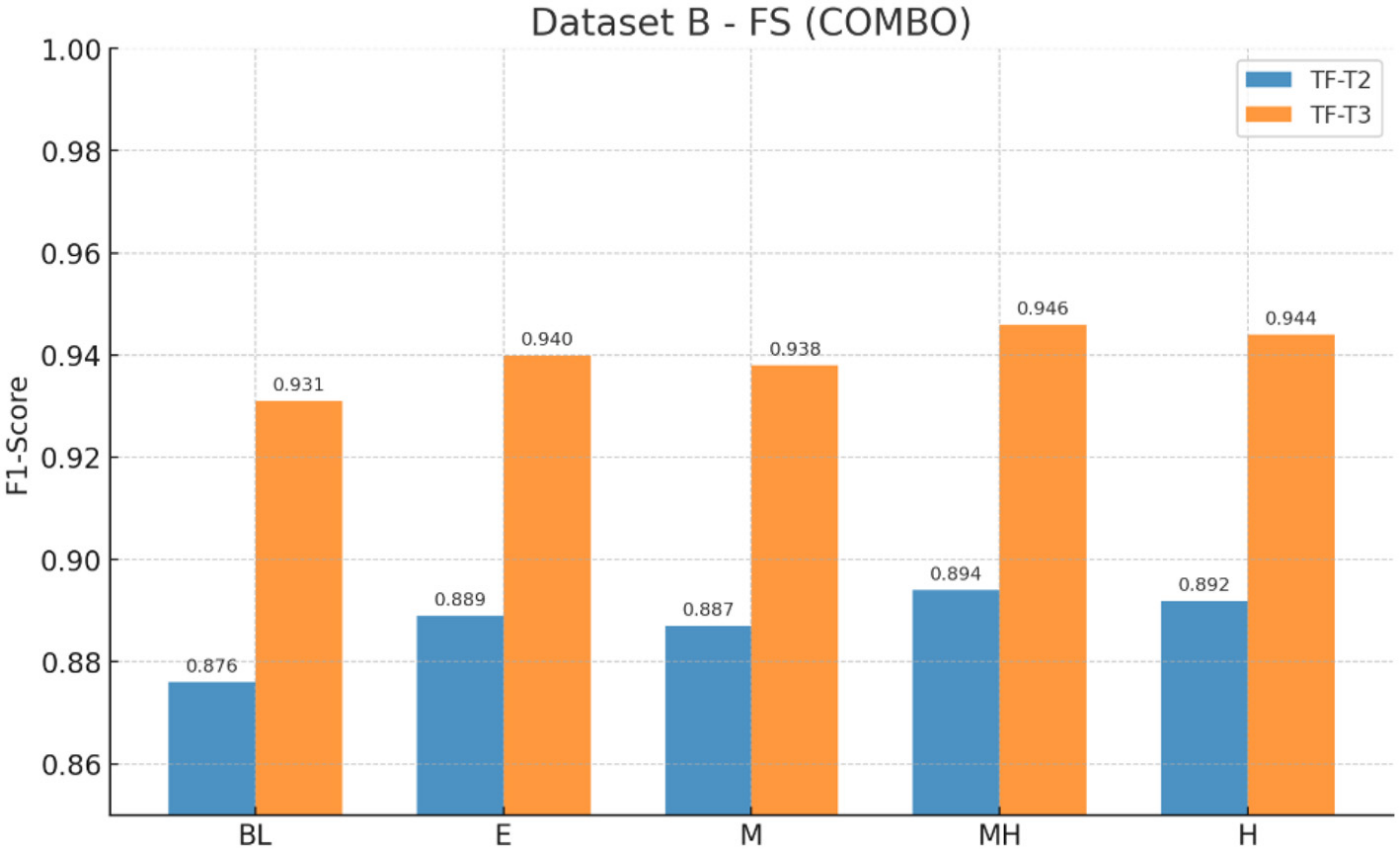
Per-class F1-score comparison between TF-T2 and TF-T3 for Dataset B/FS under COMBO noise. Gains are largest for MH and H classes, corresponding to the most complex scenarios.

Key Observations:

1. Noise-Robust Performance Gains Across All Datasets: Under COMBO noise, TF-T3 outperforms TF-T2 by +4.5 points Macro-F1 on Dataset A and +4.6 points on Dataset B, with stability coefficients reduced by ≈35%.

2. Subdivision-Level Trends (Figures 5–7): - *N-back*: Largest gain in HW (+4.8 points F1), reflecting stronger modeling of high-load working memory states. - *HtC*: Uniform improvements across all classes (≈+4.7 points). - *FS*: Largest gains in MH (+5.4 points) and H (+5.2 points), corresponding to the most complex operational scenarios.

3. Consistency Across Noise Types: Although we focus on COMBO here, TF-T3 also leads under *GAUSS* and *RAND* noise for all datasets, with absolute Macro-F1 improvements of +4.5–5.0 points over TF-T2.

4. Superiority Over Feature-Based and Alternative Architectures: Compared to the best Riemannian ensemble baseline (Alreshidi et al., 2023; Binias et al., 2018), TF-T3 improves Macro-F1 by +7–8 points in COMBO and maintains superior temporal stability, confirming that architectural advances–TF-T3's enhanced temporal–spectral representation learning and multi-resolution processing appear particularly effective in maintaining performance under mixed perturbations.

5. Deployment Relevance: The COMBO scenario is the most challenging mixed-noise evaluation condition for in-flight EEG monitoring, as it simulates the simultaneous presence of multiple noise types. TF-T3's ability to sustain high accuracy and stability in this setting underscores its readiness for operational deployment.

### 5.16 Discussion

This study presents a systematic investigation into EEG-based cognitive state classification for aviation contexts, integrating methodological innovations, rigorous evaluation protocols, and extensive robustness testing. By jointly analyzing the dataset characteristics, model design choices, experimental findings, and comparative outcomes, several key themes emerge regarding performance determinants, practical viability, and broader implications.

### 5.17 Dataset considerations and ecological validity

Two complementary datasets–Dataset A (Reducing Commercial Aviation Fatalities) and Dataset B (Flight-Deck Mental Workload)–differ in electrode density, sampling rate, and operational realism. Dataset A's high-density 22-channel EEG offers rich spatial resolution and controlled elicitation of attentional and affective states, while Dataset B's portable 14-channel system captures ecologically relevant workload variations across diverse aviation scenarios. Chronological partitioning mitigates temporal leakage, while noise-augmented variants (GAUSS, RAND, COMBO) emulate real-world perturbations. The COMBO setting, combining amplitude-dependent and amplitude-independent noise, is especially valuable for operational readiness testing.

### 5.18 Architectural contributions and design rationale

The TF-T architecture evolves through three variants (TF-T1, TF-T2, TF-T3), progressively enhancing representational capacity, attention complexity, and regularization. TF-T3 introduces additional representational capacity, multi-resolution temporal processing, and enhanced temporal–spectral interaction mechanisms aimed at improving robustness under noisy conditions. Weighted loss functions consistently mitigate class imbalance.

### 5.19 Performance trends: clean vs. noisy conditions

Under clean conditions, TF-T2 achieves the highest observed accuracy (99.2% on Dataset A) with the best FLOPs–latency trade-off, while TF-T3 does not surpass it due to mild over-parameterization. Under noise, TF-T3 consistently outperforms TF-T2 by ~4.5–4.7 Macro-F1 points across GAUSS, RAND, and COMBO, with widening gaps at higher noise levels, evidencing superior robustness to spectral drift and transient channel corruption.

### 5.20 Class-wise sensitivity

Per-class results show that TF-T2 dominates on clean data, with TF-T3 narrowing the gap for transient, phasic states (e.g., SS in Dataset A). In Dataset B, TF-T3 gains are concentrated in higher workload classes (HW, MH, H), which present overlapping temporal–spectral profiles that benefit from multi-resolution and cross-domain modeling.

### 5.21 Comparative standing against state-of-the-art

Against Riemannian geometry-based classifiers and deep learning baselines, TF-T3 delivers superior Macro-F1 and temporal stability under COMBO noise, with margins up to +8 points over the best feature-based ensemble. These improvements are attributable to the combined effects of multi-resolution attention, temporal–spectral integration, and explicit modeling of state dynamics.

### 5.22 Computational considerations

TF-T3's complexity increases inference time by ≈47% relative to TF-T2, reducing throughput from 189 to 147 epochs/s. The trade-off is justified in high-perturbation environments where robustness is paramount, but TF-T2 remains preferable for efficiency-critical, low-noise monitoring.

### 5.23 Error taxonomy and decision boundaries

Error analysis shows that most confusions occur between adjacent cognitive states (e.g., DA → CA; M↔MH), with TF-T3 under noise shifting misclassifications toward such benign neighbors rather than distant classes–indicating robustness via representation smoothing rather than indiscriminate compression.

### 5.24 Limitations and future directions

Low-support classes in Dataset B/FS yield wider confidence intervals, modestly increasing per-class estimate variance. COMBO noise captures multiple perturbation types but does not encompass all in-flight interference patterns (e.g., persistent channel dropout). Future work should integrate adaptive thresholding, cost-sensitive training, and multimodal sensor fusion to further enhance resilience. Although the adopted perturbation protocol captures several common forms of signal corruption, it does not explicitly model all aviation-relevant EEG artifacts, including electrode displacement, motion-related contamination, persistent channel dropout, or impedance fluctuations.

#### 5.24.1 Key takeaways

1. Dataset Complementarity and Ecological Validity: The dual-dataset design enables both upper-bound performance assessment (Dataset A) and deployment-relevant robustness evaluation (Dataset B). Chronological partitioning and noise-augmented variants ensure operational realism.
2. Architecture–Performance Trade-offs: TF-T2 offers the optimal clean-data accuracy–efficiency balance; TF-T3's higher complexity is justified under noise, yielding ~4.5–4.7 point Macro-F1 gains across perturbation types.
3. Noise-Robustness Mechanisms: Enhanced temporal–spectral integration and multi-resolution processing contribute to improved robustness under mixed-noise conditions.
4. Class-Specific Sensitivity: Gains are most pronounced in high-workload classes, improving separability without compromising calibration.
5. Comparative Superiority: TF-T3 outperforms Riemannian geometry-based ensembles and deep learning baselines by +7–8 Macro-F1 points under COMBO noise while reducing temporal instability by ≈35%.
6. Deployment Guidance: TF-T2 is preferred for efficiency-critical low-noise monitoring; TF-T3 is recommended for perturbation-prone environments prioritizing robustness.

## 6 Conclusions

This study advances EEG-based cognitive state classification for aviation by introducing a noise-robust TF-T architecture. Leveraging complementary datasets, we demonstrate high accuracy under controlled conditions and strong robustness in ecologically realistic, noise-augmented scenarios. TF-T2 offers the best accuracy–efficiency trade-off for clean data, while TF-T3, incorporating enhanced temporal–spectral integration and multi-resolution temporal processing, consistently outperforms alternative approaches under noisy conditions. Limitations include wider confidence intervals for low-support classes, incomplete coverage of real-world interference patterns, and increased inference latency (≈47%) for TF-T3. Future work will address these by exploring adaptive decision thresholding, cost-sensitive training to mitigate class imbalance, and multimodal sensor fusion (e.g., EEG with eye-tracking or physiological measures) to enhance resilience. Additionally, incorporating structured artifact simulations and optimizing transformer efficiency will further support scalable, real-time deployment in cockpit monitoring systems.

## Data Availability

Publicly available datasets were analyzed in this study. This data can be found here: https://www.kaggle.com/competitions/reducing-commercial-aviation-fatalities.
